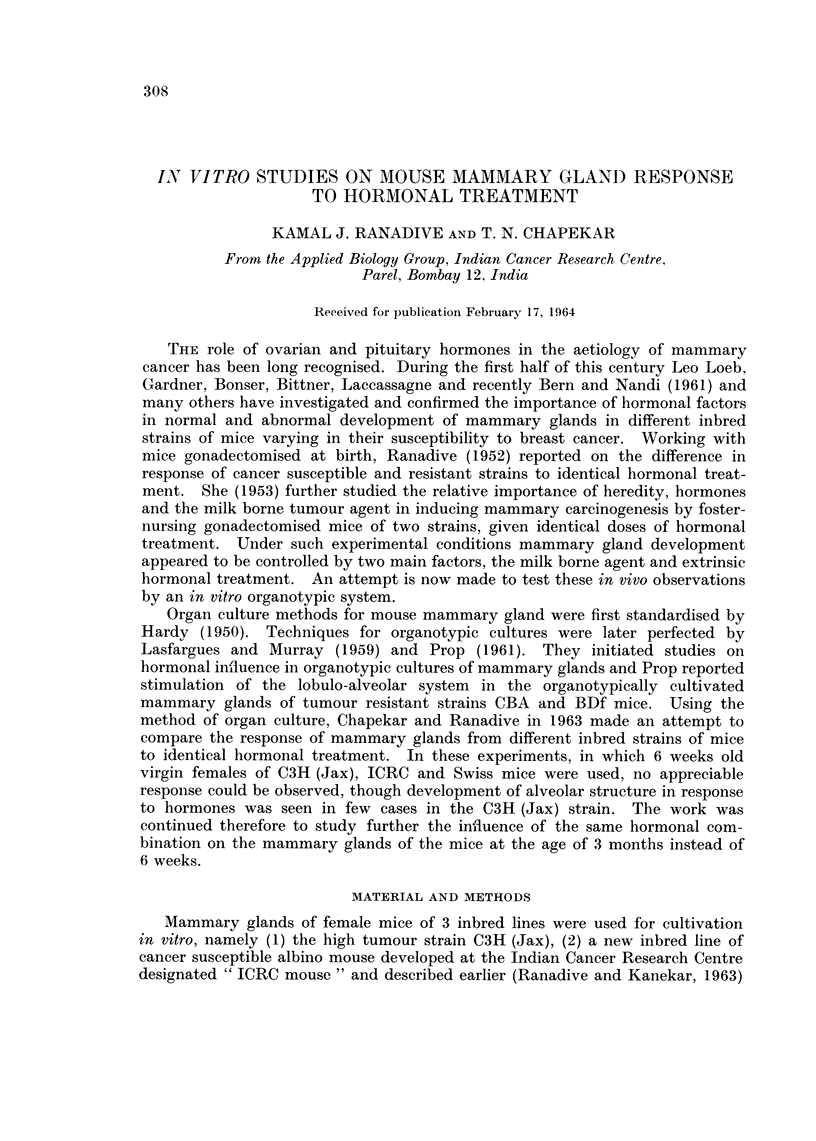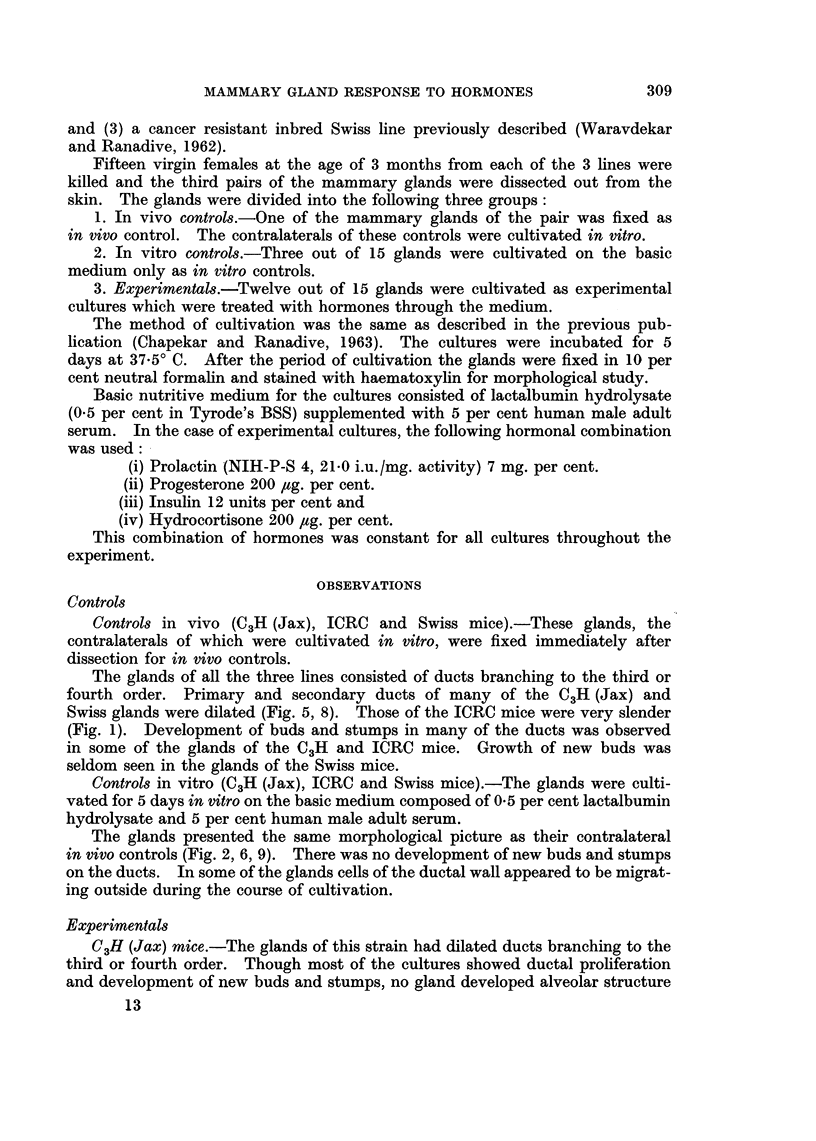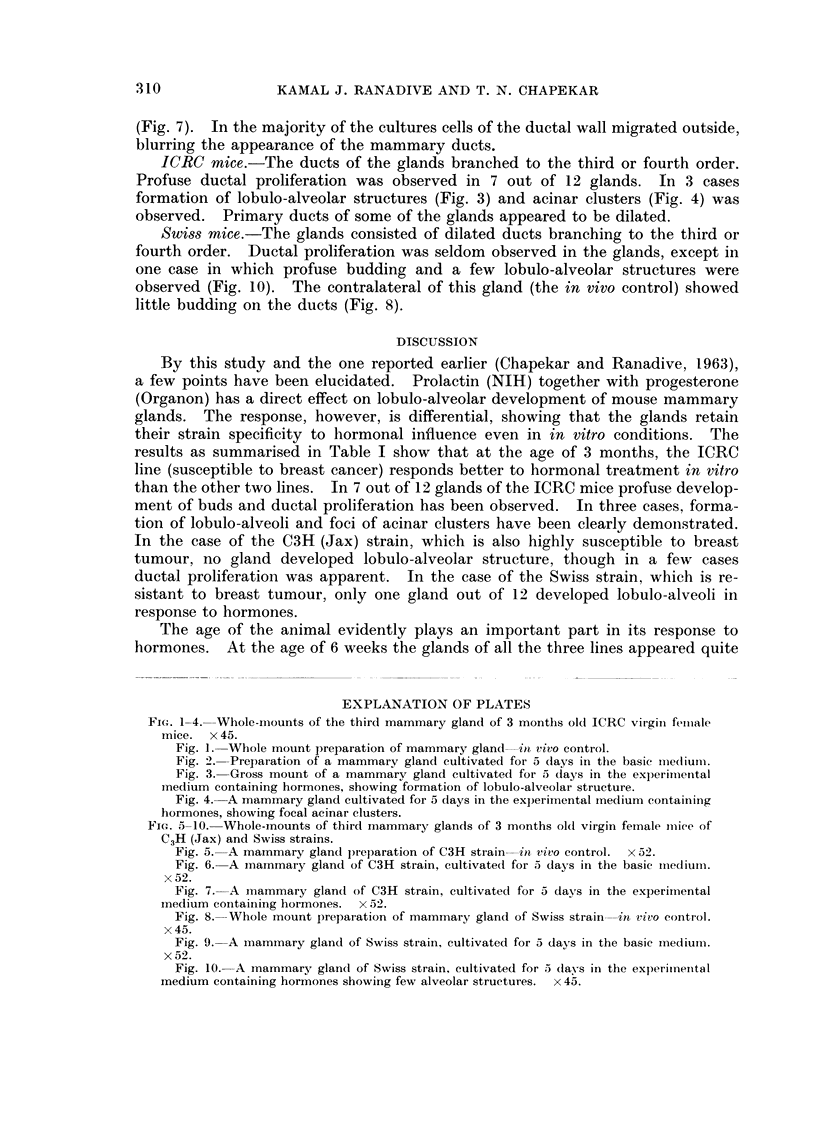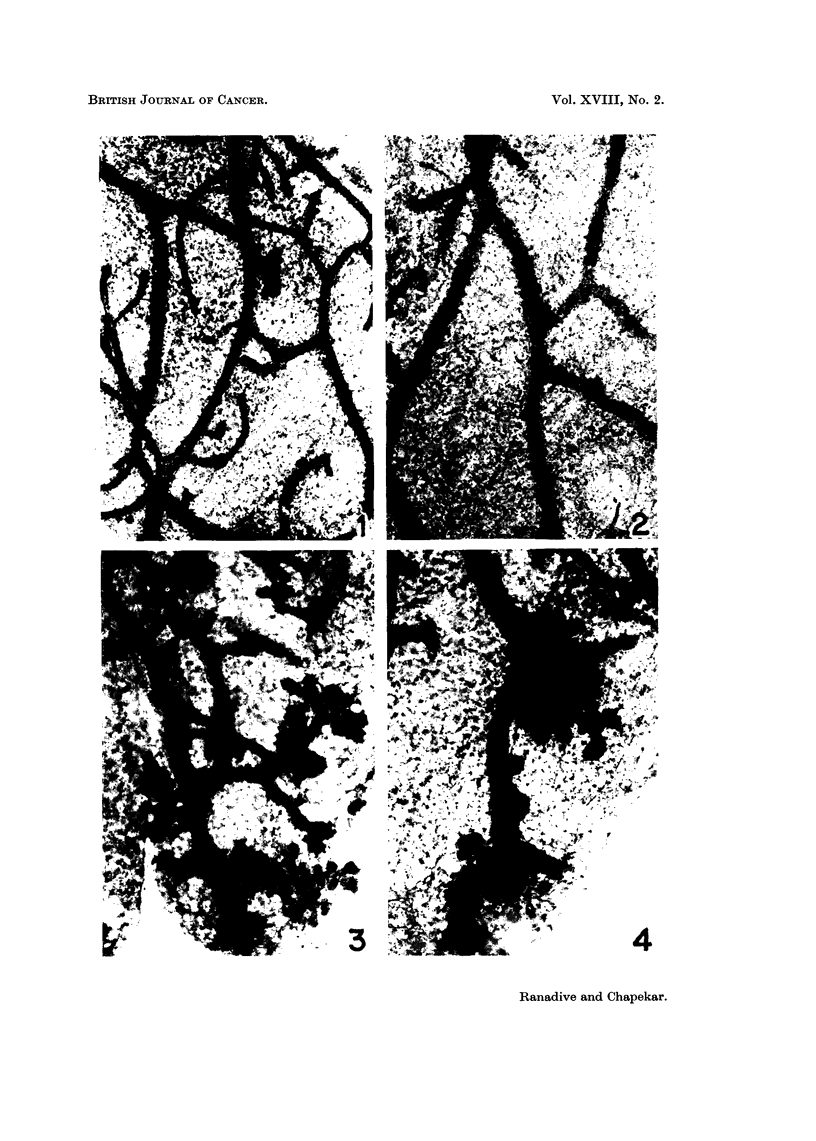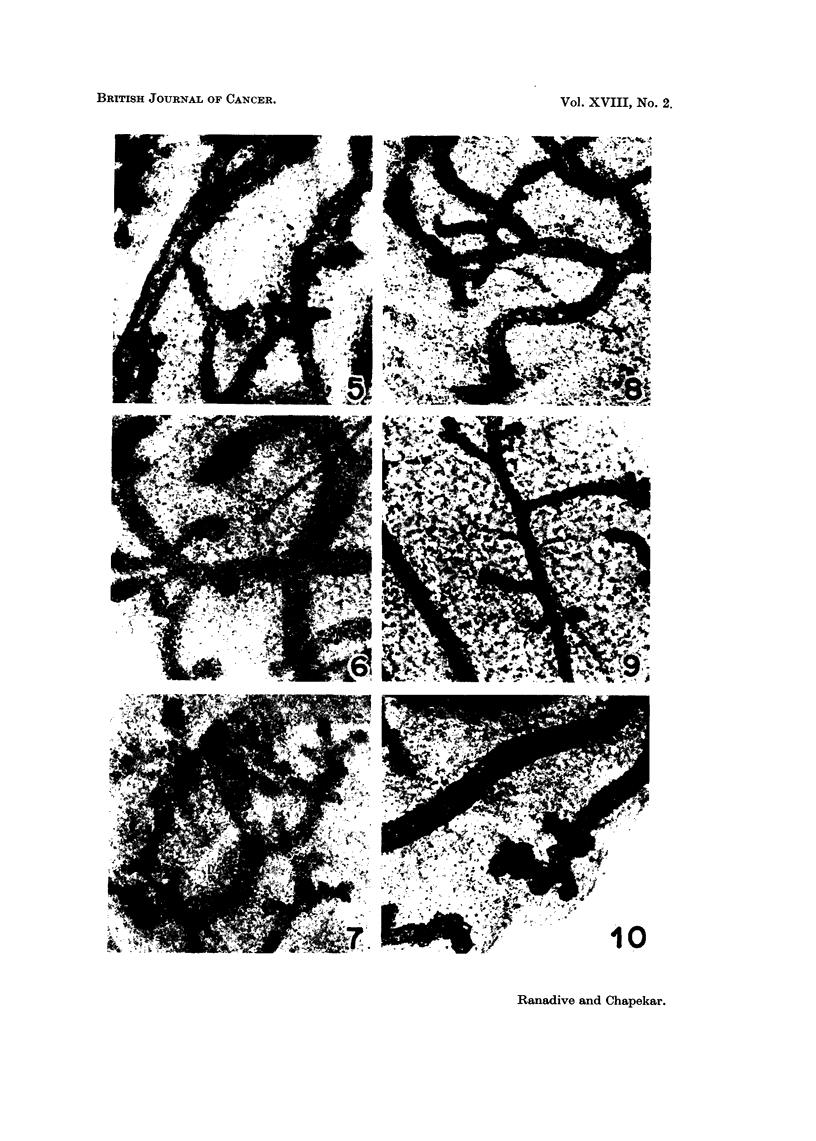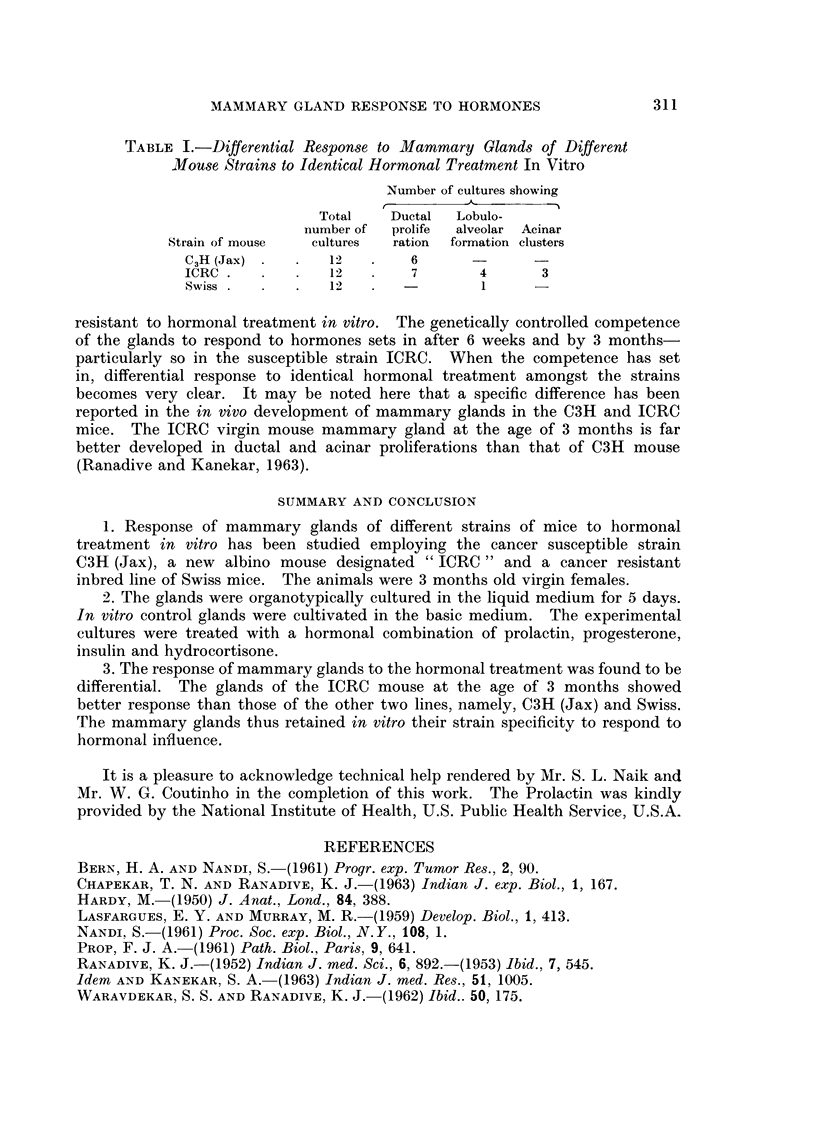# In Vitro Studies on Mouse Mammary Gland Response to Hormonal Treatment

**DOI:** 10.1038/bjc.1964.35

**Published:** 1964-06

**Authors:** Kamal J. Ranadive, T. N. Chapekar

## Abstract

**Images:**


### *IN VITRO* STUDIES ON MOUSE MAMMARY GLAND RESPONSE TO HORMONAL TREATMENT

KAMAL J. RANADIVE AND T. N. CHAPEKAR

*From the Applied Biology Group, Indian Cancer Research Centre, Parel, Bombay 12, India*

Received for publication February 17, 1964

THE role of ovarian and pituitary hormones in the aetiology of mammary cancer has been long recognised. During the first half of this century Leo Loeb, Gardner, Bonser, Bittner, Laccassagne and recently Bern and Nandi (1961) and many others have investigated and confirmed the importance of hormonal factors in normal and abnormal development of mammary glands in different inbred strains of mice varying in their susceptibility to breast cancer. Working with mice gonadectomised at birth, Ranadive (1952) reported on the difference in response of cancer susceptible and resistant strains to identical hormonal treatment. She (1953) further studied the relative importance of heredity, hormones and the milk borne tumour agent in inducing mammary carcinogenesis by foster-nursing gonadectomised mice of two strains, given identical doses of hormonal treatment. Under such experimental conditions mammary gland development appeared to be controlled by two main factors, the milk borne agent and extrinsic hormonal treatment. An attempt is now made to test these *in vivo* observations by an *in vitro* organotypic system.

Organ culture methods for mouse mammary gland were first standardised by Hardy (1950). Techniques for organotypic cultures were later perfected by Lasfargues and Murray (1959) and Prop (1961). They initiated studies on hormonal influence in organotypic cultures of mammary glands and Prop reported stimulation of the lobulo-alveolar system in the organotypically cultivated mammary glands of tumour resistant strains CBA and BDf mice. Using the method of organ culture, Chapekar and Ranadive in 1963 made an attempt to compare the response of mammary glands from different inbred strains of mice to identical hormonal treatment. In these experiments, in which 6 weeks old virgin females of C3H (Jax), ICRC and Swiss mice were used, no appreciable response could be observed, though development of alveolar structure in response to hormones was seen in few cases in the C3H (Jax) strain. The work was continued therefore to study further the influence of the same hormonal combination on the mammary glands of the mice at the age of 3 months instead of 6 weeks.

### MATERIAL AND METHODS

Mammary glands of female mice of 3 inbred lines were used for cultivation *in vitro*, namely (1) the high tumour strain C3H (Jax), (2) a new inbred line of cancer susceptible albino mouse developed at the Indian Cancer Research Centre designated " ICRC mouse " and described earlier (Ranadive and Kanekar, 1963)

and (3) a cancer resistant inbred Swiss line previously described (Waravdekar and Ranadive, 1962).

Fifteen virgin females at the age of 3 months from each of the 3 lines were killed and the third pairs of the mammary glands were dissected out from the skin. The glands were divided into the following three groups :

1. In vivo *controls.*—One of the mammary glands of the pair was fixed as *in vivo* control. The contralaterals of these controls were cultivated *in vitro.*

2. In vitro *controls.*—Three out of 15 glands were cultivated on the basic medium only as *in vitro* controls.

3. *Experimentals.*—Twelve out of 15 glands were cultivated as experimental cultures which were treated with hormones through the medium.

The method of cultivation was the same as described in the previous publication (Chapekar and Ranadive, 1963). The cultures were incubated for 5 days at 37·5° C. After the period of cultivation the glands were fixed in 10 per cent neutral formalin and stained with haematoxylin for morphological study.

Basic nutritive medium for the cultures consisted of lactalbumin hydrolysate (0·5 per cent in Tyrode's BSS) supplemented with 5 per cent human male adult serum. In the case of experimental cultures, the following hormonal combination was used :

(i) Prolactin (NIH-P-S 4, 21·0 i.u./mg. activity) 7 mg. per cent.
(ii) Progesterone 200 $\mu$g. per cent.
(iii) Insulin 12 units per cent and
(iv) Hydrocortisone 200 $\mu$g. per cent.

This combination of hormones was constant for all cultures throughout the experiment.

### OBSERVATIONS

### *Controls*

*Controls* in vivo ($C_3H$ (Jax), ICRC and Swiss mice).—These glands, the contralaterals of which were cultivated *in vitro*, were fixed immediately after dissection for *in vivo* controls.

The glands of all the three lines consisted of ducts branching to the third or fourth order. Primary and secondary ducts of many of the $C_3H$ (Jax) and Swiss glands were dilated (Fig. 5, 8). Those of the ICRC mice were very slender (Fig. 1). Development of buds and stumps in many of the ducts was observed in some of the glands of the $C_3H$ and ICRC mice. Growth of new buds was seldom seen in the glands of the Swiss mice.

*Controls* in vitro ($C_3H$ (Jax), ICRC and Swiss mice).—The glands were cultivated for 5 days *in vitro* on the basic medium composed of 0·5 per cent lactalbumin hydrolysate and 5 per cent human male adult serum.

The glands presented the same morphological picture as their contralateral *in vivo* controls (Fig. 2, 6, 9). There was no development of new buds and stumps on the ducts. In some of the glands cells of the ductal wall appeared to be migrating outside during the course of cultivation.

### *Experimentals*

*$C_3H$ (Jax) mice.*—The glands of this strain had dilated ducts branching to the third or fourth order. Though most of the cultures showed ductal proliferation and development of new buds and stumps, no gland developed alveolar structure

(Fig. 7). In the majority of the cultures cells of the ductal wall migrated outside, blurring the appearance of the mammary ducts.

*ICRC mice.*—The ducts of the glands branched to the third or fourth order. Profuse ductal proliferation was observed in 7 out of 12 glands. In 3 cases formation of lobulo-alveolar structures (Fig. 3) and acinar clusters (Fig. 4) was observed. Primary ducts of some of the glands appeared to be dilated.

*Swiss mice.*—The glands consisted of dilated ducts branching to the third or fourth order. Ductal proliferation was seldom observed in the glands, except in one case in which profuse budding and a few lobulo-alveolar structures were observed (Fig. 10). The contralateral of this gland (the *in vivo* control) showed little budding on the ducts (Fig. 8).

### DISCUSSION

By this study and the one reported earlier (Chapekar and Ranadive, 1963), a few points have been elucidated. Prolactin (NIH) together with progesterone (Organon) has a direct effect on lobulo-alveolar development of mouse mammary glands. The response, however, is differential, showing that the glands retain their strain specificity to hormonal influence even in *in vitro* conditions. The results as summarised in Table I show that at the age of 3 months, the ICRC line (susceptible to breast cancer) responds better to hormonal treatment *in vitro* than the other two lines. In 7 out of 12 glands of the ICRC mice profuse development of buds and ductal proliferation has been observed. In three cases, formation of lobulo-alveoli and foci of acinar clusters have been clearly demonstrated. In the case of the C3H (Jax) strain, which is also highly susceptible to breast tumour, no gland developed lobulo-alveolar structure, though in a few cases ductal proliferation was apparent. In the case of the Swiss strain, which is resistant to breast tumour, only one gland out of 12 developed lobulo-alveoli in response to hormones.

The age of the animal evidently plays an important part in its response to hormones. At the age of 6 weeks the glands of all the three lines appeared quite

---

### EXPLANATION OF PLATES

FIG. 1–4.—Whole-mounts of the third mammary gland of 3 months old ICRC virgin female mice. ×45.

Fig. 1.—Whole mount preparation of mammary gland—*in vivo* control.

Fig. 2.—Preparation of a mammary gland cultivated for 5 days in the basic medium.

Fig. 3.—Gross mount of a mammary gland cultivated for 5 days in the experimental medium containing hormones, showing formation of lobulo-alveolar structure.

Fig. 4.—A mammary gland cultivated for 5 days in the experimental medium containing hormones, showing focal acinar clusters.

FIG. 5–10.—Whole-mounts of third mammary glands of 3 months old virgin female mice of $C_3H$ (Jax) and Swiss strains.

Fig. 5.—A mammary gland preparation of C3H strain—*in vivo* control. ×52.

Fig. 6.—A mammary gland of C3H strain, cultivated for 5 days in the basic medium. ×52.

Fig. 7.—A mammary gland of C3H strain, cultivated for 5 days in the experimental medium containing hormones. ×52.

Fig. 8.—Whole mount preparation of mammary gland of Swiss strain—*in vivo* control. ×45.

Fig. 9.—A mammary gland of Swiss strain, cultivated for 5 days in the basic medium. ×52.

Fig. 10.—A mammary gland of Swiss strain, cultivated for 5 days in the experimental medium containing hormones showing few alveolar structures. ×45.

Ranadive and Chapekar.

Ranadive and Chapekar.

TABLE I.—*Differential Response to Mammary Glands of Different Mouse Strains to Identical Hormonal Treatment* In Vitro

| Strain of mouse | Total number of cultures | Number of cultures showing: Ductal proliferation | Lobulo-alveolar formation | Acinar clusters |
|---|---|---|---|---|
| $C_3H$ (Jax) | 12 | 6 | — | — |
| ICRC | 12 | 7 | 4 | 3 |
| Swiss | 12 | — | 1 | — |

resistant to hormonal treatment *in vitro.* The genetically controlled competence of the glands to respond to hormones sets in after 6 weeks and by 3 months—particularly so in the susceptible strain ICRC. When the competence has set in, differential response to identical hormonal treatment amongst the strains becomes very clear. It may be noted here that a specific difference has been reported in the *in vivo* development of mammary glands in the C3H and ICRC mice. The ICRC virgin mouse mammary gland at the age of 3 months is far better developed in ductal and acinar proliferations than that of C3H mouse (Ranadive and Kanekar, 1963).

### SUMMARY AND CONCLUSION

1. Response of mammary glands of different strains of mice to hormonal treatment *in vitro* has been studied employing the cancer susceptible strain C3H (Jax), a new albino mouse designated " ICRC " and a cancer resistant inbred line of Swiss mice. The animals were 3 months old virgin females.

2. The glands were organotypically cultured in the liquid medium for 5 days. *In vitro* control glands were cultivated in the basic medium. The experimental cultures were treated with a hormonal combination of prolactin, progesterone, insulin and hydrocortisone.

3. The response of mammary glands to the hormonal treatment was found to be differential. The glands of the ICRC mouse at the age of 3 months showed better response than those of the other two lines, namely, C3H (Jax) and Swiss. The mammary glands thus retained *in vitro* their strain specificity to respond to hormonal influence.

It is a pleasure to acknowledge technical help rendered by Mr. S. L. Naik and Mr. W. G. Coutinho in the completion of this work. The Prolactin was kindly provided by the National Institute of Health, U.S. Public Health Service, U.S.A.

### REFERENCES

BERN, H. A. AND NANDI, S.—(1961) *Progr. exp. Tumor Res.*, **2**, 90.
CHAPEKAR, T. N. AND RANADIVE, K. J.—(1963) *Indian J. exp. Biol.*, **1**, 167.
HARDY, M.—(1950) *J. Anat., Lond.*, **84**, 388.
LASFARGUES, E. Y. AND MURRAY, M. R.—(1959) *Develop. Biol.*, **1**, 413.
NANDI, S.—(1961) *Proc. Soc. exp. Biol., N.Y.*, **108**, 1.
PROP, F. J. A.—(1961) *Path. Biol., Paris*, **9**, 641.
RANADIVE, K. J.—(1952) *Indian J. med. Sci.*, **6**, 892.—(1953) *Ibid.*, **7**, 545.
*Idem* AND KANEKAR, S. A.—(1963) *Indian J. med. Res.*, **51**, 1005.
WARAVDEKAR, S. S. AND RANADIVE, K. J.—(1962) *Ibid.*. **50**, 175.